# The effects of dispersal, herbivory, and competition on plant community assembly

**DOI:** 10.1002/ecy.3859

**Published:** 2022-11-10

**Authors:** Samantha A. Allbee, Haldre S. Rogers, Lauren L. Sullivan

**Affiliations:** ^1^ Department of Ecology, Evolution, and Organismal Biology (EEOB) Iowa State University Ames Iowa USA; ^2^ Division of Biological Sciences University of Missouri Columbia Missouri USA; ^3^ Department of Plant Biology Michigan State University East Lansing Michigan USA

**Keywords:** biotic interactions, competition, restoration experiment, seed dispersal, species diversity, trade‐offs

## Abstract

Dispersal is a key process in community assembly but is often considered separately from downstream assembly processes (e.g., competition, herbivory). However, dispersal varies by species and can interact with other assembly processes through establishment as species enter communities. Here, we sought to distinguish the role of dispersal in community assembly and its interaction with two biotic assembly processes: competition and herbivory. We used a tallgrass prairie restoration experiment that manipulated the competitive and herbivore environments while allowing for natural dispersal and establishment from a diverse regional species pool into areas of low diversity. Dispersal, competition, and herbivory all influenced local communities. By tracking the spread of four target species across the plots, we found interspecific and intraspecific differences in establishment patterns, with herbivores influencing the number of individuals present and the distances species moved. At the community level, only dispersal and competition significantly influenced alpha diversity, but all three processes additively influenced community composition. There was also evidence of herbivore–competition and herbivore–colonization trade‐offs in our experiment. Some species that could tolerate herbivory were less likely to establish in competitive environments, while others that could tolerate herbivory were more likely to disperse greater distances. More work is needed to understand the contexts under which dispersal variation affects community assembly and its synergy with other processes.

## INTRODUCTION

Community assembly provides a conceptual foundation for the processes that determine which and how many species exist in a locality (Chase, 2003; Samuels & Drake, 1997). A fundamental goal in community ecology is to identify the processes that select for or against a species in the regional species pool, which ultimately determines the local community composition (HilleRisLambers et al., 2012; Keddy, 1992). Although the idea of filtering species from the regional species pool into local communities has existed in a linear, sieve‐like fashion for decades (i.e., species disperse, then experience abiotic filtering, and then interact with the local biotic community) (Keddy, 1992), recent work has demonstrated that more complexity exists in assembly through feedbacks between these processes (HilleRisLambers et al., 2012; Kraft et al., 2015). For example, under some conditions, abiotic and biotic processes are not sequential but instead interact to alter the outcomes of survival and establishment in local communities (Germain et al., 2019). Although there has been a push to unpack the complexities of other abiotic and biotic assembly processes, less attention has been paid to dispersal and how it interacts with other processes.

A critical, but often simplified, first step to community assembly is dispersal, where propagules from the regional species pool disperse to local communities before they begin interacting with the abiotic and biotic environments in their new locations (Vellend, 2010). Many community assembly studies consider dispersal to be a binary process, where seeds either do or do not make it to a particular location (e.g., Kraft et al., 2015). Indeed, seed addition studies largely show that when propagules of absent species are added to a community to overcome dispersal limitation, diversity increases (Clark et al., 2007). However, in naturally establishing communities, dispersal is more complicated. Instead of assuming dispersal as a binary arrival process, probability distributions are more appropriately used to describe plant movement (Bullock et al., 2017) and usually convey the reality that more propagules will predictably arrive nearer to source locations and fewer propagules will move longer distances. Accordingly, more recent experimental results demonstrate that the degree of dispersal limitation varies with distance from a source population (Germain et al., 2017; Pinto & MacDougall, 2010). Additionally, to become part of a community, a propagule must arrive, germinate, emerge, and establish (termed “effective dispersal”; Nathan, 2013). Thus, the community present at a given location is the result of effective dispersal and other interacting processes. Other assembly processes intrinsically affect both dispersal and establishment and, thus, must be considered jointly when teasing apart the role of dispersal during assembly. A more nuanced understanding of dispersal will more accurately reflect the assembly processes individuals experience because it reflects the interacting nature of assembly.

Biotic assembly processes can interact with dispersal to alter local communities. In this work, we focus on these two biotic processes, herbivory and competition, because they affect establishment, even though dispersal likely interacts with the abiotic environment as well (Muthukrishnan et al., 2020; Teller et al., 2014). Herbivores may alter dispersal distance directly, through ecto‐ or endozoochory or caching, which tends to increase dispersal distance (Benton, 2012), or indirectly, by altering plant height through consumption, which can decrease dispersal distance (Thomson et al., 2011). Once seeds have arrived, selective foraging by herbivores on either seeds or established individuals could modify or even erase the signal of dispersal, affecting spatial patterns of assembly (Howe et al., 2002; Howe & Brown, 2000). Therefore, herbivores may have a strong influence in shaping plant communities both alone and through interactions with dispersal and then establishment.

Dispersal may also interact with competition since seeds that arrive at a location must also establish. For example, variation in the timing and abundance of seed arrival can impact competition via priority effects (Fukami, 2015). Through this form of competition, existing species or communities may inhibit (or facilitate) the establishment of newly arriving species. Although theoretical support for the interaction of dispersal and competition is strong (i.e., the competition–colonization trade‐off), empirical tests in communities are less common (Bolker et al., 2003; Levine & Murrell, 2003). Interactions among these assembly processes (e.g., dispersal, herbivory, and competition) may in fact be the norm, and experimentation is necessary to disentangle when and where specific processes drive community assembly (Going et al., 2009; Kraft et al., 2015).

Species' additive or interacting responses to processes during assembly likely result in species' trade‐offs. Experiments tend to demonstrate trade‐offs among these processes. Plants that are stronger competitors are often less tolerant of herbivores (Kneitel & Chase, 2004), and species that are better able to tolerate stress (e.g., herbivory, competition, abiotic conditions) are often poorer colonizers (Muller‐Landau, 2010). However, empirical evidence for competition–defense and competition–colonization trade‐offs in plants is limited, which results in uncertainty of the mechanisms by which competitors and consumers regulate assembly (Viola et al., 2010). Thus, exploring how individual species solve the challenges of reaching new areas, tolerating herbivory, competing with the local community, and successfully establishing can lead to greater understanding of how trade‐offs help structure assembly (Weiher & Keddy, 1999).

In this study, we used an experimental grassland restoration to explore the role of dispersal, herbivory, and competition in community assembly. Our experimental site consists of eight plots that contain a high‐diversity central core seeded with 51 species and a low‐diversity exterior matrix seeded with a subset of 14 species from the core (see subsequent discussion). To assess dispersal, we evaluated the movement of plants away from the central core. To assess the role of competition, four cover strips were added in each plot before seeding to prevent colonization and then removed 2 years later to allow colonization by natural dispersal from the core and matrix. Comparisons between quadrats near and far from the central core allowed us to compare the influence of dispersal in areas with high and low levels of competition. To test the impact of herbivores on assembly processes, we fenced half the plots to exclude most mammalian herbivores. We pose the following questions:

1. Are there inter‐ and intraspecific differences in effective dispersal in our communities that assemble under different herbivore conditions?
2. How do the processes of dispersal, competition, and herbivory alter community assembly at the individual and community level?
3. Do we see evidence of competition–colonization, herbivory–colonization, and herbivory–competition trade‐offs in our assembly experiment?

## METHODS

### Study site and experimental design

**FIGURE 1:**
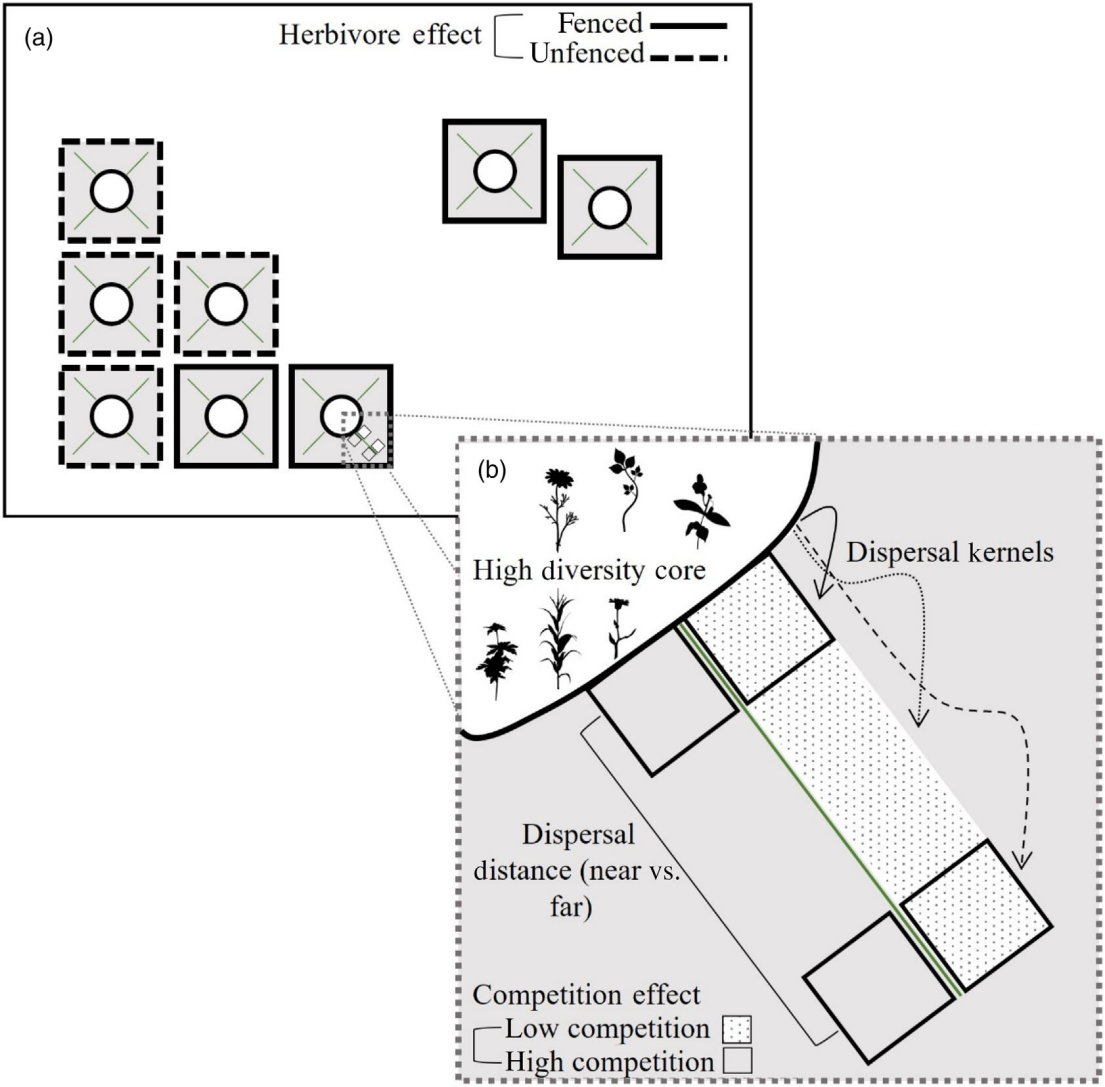
Study site layout of Oakridge prairie. (a) Site layout of all eight plots. The circles inside each plot represent the high‐diversity cores initially seeded with 51 species. Outside of the cores within each plot is the matrix, initially seeded with 14 species (a subset of the high‐diversity species). Lines radiating from the cores within each plot represent the 1.8 × 10‐m^2^ transects where we sampled plant communities. Plots with solid outlines are fenced and exclude herbivory, and plots with dotted outlines allow herbivores access. (b) Zoomed‐in view of plot to demonstrate sampling scheme. The white dotted area represents the transect that was covered at the time of initial seeding and exposed to seed input 2 years later, while the gray area depicts the region seeded with the matrix species. Each transect has two 1‐m^2^ quadrats—one close to the core and one far from the core—where we sampled the plant community. The comparison between quadrats in covered and uncovered transects allows us to determine the competition effect. The comparison between near and far quadrats tests the importance of dispersal limitation. Herbivory effects are determined by comparing plots with and without herbivore fences.

Oakridge Research and Education Prairie is a 1.6‐ha tallgrass prairie restoration in Ames, Iowa, on land that was originally managed for alternating corn and soy crops for at least 100 years before tilling and seeding in March of 2012 (for a full methods description see Mortensen et al. [2013] and Appendix S1). Within Oakridge Prairie, we created eight experimental plots (hereafter termed “plots”), each strategically seeded to facilitate tracking the movement of plant species (Figure 1a). In each plot, we seeded a circular central core area 19.2 m in diameter (hereafter termed “core”) with 51 plant species creating a region with high plant diversity. We seeded the remainder of each 32 × 32‐m square plot with 14 plant species, which were a subset of the 51 species seeded in the core (hereafter termed “matrix”). Thus, the core contained 37 unique species (i.e., not seeded elsewhere), and we assumed any occurrence of these species in the matrix to be the result of effective dispersal. None of these 37 species spread significant distances by rhizomes, and we saw no evidence of these species on the landscape other than from our seeding—likely due to long‐term local extirpation from conversion to agriculture.

In this study, we focused on the established plant communities, and thus, our measure of dispersal is a measure of effective dispersal or the combination of movement and establishment (Nathan, 2013). Effective dispersal is the positive signature of dispersal—it represents those seeds that both arrived and survived through establishment. We did not measure seed arrival, which would have been a direct measure of dispersal and would likely have identified more species and individuals that arrived but did not establish. Specifically, poor competitors may not be represented in our study if they were able to disperse but not establish. However, by conducting our study within a largely homogeneous abiotic environment and experimentally controlling for biotic establishment processes of competition and herbivory, we maintain that variation in established plants along spatial gradients away from the source population largely reflects differences in movement.

Our experimental prairie restoration was designed to examine the joint effects of dispersal, competition, and herbivory on plant community assembly. First, distance from the core reflects dispersal.

Second, to explore how competition affects assembly, we created four sets of paired 1.8‐m‐wide × 10‐m‐long transects (hereafter termed “transects”) in the matrix of each experimental plot. These transects began at the edge of the core, near the high‐diversity seeding, and radiated to the corner of each plot, far from the high‐diversity seeding (Figure 1b). Before the initial seeding in 2012, we covered one transect per pair (total four transects/plot) with 4‐mil, string‐reinforced opaque plastic to prevent seed arrival. Thus, while covered, no dispersal or establishment occurred during the initial restoration. Adjacent and parallel to the covered transect, we marked the other transect of the pair; this transect was seeded during the initial restoration seeding with matrix species in 2012, producing areas with high competition relative to the unseeded covered transect. Assignment of covered versus uncovered treatment within each pair was random. We removed the plastic coverings in the fall of 2014, exposing the transect to natural dispersal from the surrounding matrix and species from the core. The open space allowed species to arrive unhindered by competition from already established species. The comparison between these paired transects allowed us to understand how competition from the restored plant community influenced assembly—covered transects had low competition, whereas uncovered transects had high competition.

Third, to determine the effects of herbivores on plant communities, we fenced four of the plots to exclude most mammalian herbivores (mainly deer and voles; Figure 1a), reduced deer abundance through an offset electric fence (Craven & Hygnstrom, 1994), and maintained low small‐mammal abundance through buried hardware cloth that extended 30 cm below ground and 50 cm above ground. We also conducted initial trapping and removal of small mammals and subsequent mowing of “fear strips” around each plot (Salmon & Gorenzel, 2010). Owing to the logistics of maintaining electrical fences and limitations of the site, the herbivore plots were set up as adjacent pairs, and two plots were not contiguous with the other six plots. Although we cannot completely isolate the influence of plot arrangement from the influence of herbivores, we did not find significant differences in the communities within the two noncontiguous plots relative to the six contiguous plots (Appendix S1). All community data were collected in 2019, allowing 5 (covered transects) or 7 years (uncovered transects) for species to move from the core into the matrix.

### Data collection

#### Target plant species and movement

In addition to our experimental design, which allowed us to examine community‐level assembly, we explored population‐level movement patterns on our landscape. To evaluate inter‐ and intraspecific variation in effective dispersal, we mapped the location of all individuals of four species—*Penstemon digitalis*, *Baptisia alba*, *Symphyotrichum ericoides*, and *Eryngium yuccifolium*—which were only seeded in the core. Therefore, any individual found outside of the core was due to dispersal and establishment. All four species are primarily passively dispersed, but their dispersal modes vary, with *P. digitalis* and *B. alba* demonstrating gravity or explosive dispersal, which is likely to result in shorter dispersal distances, and *S. ericoides* and *E. yuccifolium* possessing structures that facilitate wind dispersal and likely longer dispersal distances (Gleason & Cronquist, 1963). These species were selected because they were easily identifiable late in the season and their dispersal modes reflected a gradient of potential movement distance (Appendix S1).

To measure the specific effects of herbivores on plant species movement, we searched all of our experimental plots for all individuals of our four target species: *S. ericoides*, *B. alba*, *E. yuccifolium*, and *P. digitalis*. Although *S. ericoides* can be clonal, we did not find multiple stems in a single location (i.e., no clonal growth by rhizomes), so we considered each stem to be an individual for the target species. We then used a Trimble Geo 7X to record the exact location of each individual to the nearest 10 cm (Appendix S1: Figure S1). Since these species were all seeded in the core, we excluded all individuals within the core from analyses. Any individual that was found outside the core was a result of effective dispersal; thus, we calculated the distance from the edge of the core for each individual to measure the establishment distance per species per plot. We then combined distances for species separately for herbivore and no‐herbivore plots to create “establishment kernels” to reflect the joint spatial processes of dispersal and establishment on the landscape.

#### Community composition

To quantify how dispersal distance, herbivory, and competition altered plant species diversity and community composition in the restored prairie, we estimated species cover in paired 1‐m^2^ quadrats (hereafter termed “quadrats”) using the Daubenmeyer method. We surveyed two 1‐m^2^ quadrats within each of the paired transects (Figure 1b). Half of the quadrats were “near” (adjacent to the high‐diversity core, which represented a short dispersal distance), and half were “far” quadrats (>9 m away from the core and represented a far dispersal distance). Within each set of transects (covered–low competition and uncovered–high competition), we sampled the near and far 1‐m^2^ quadrats to examine how the distance from the core influenced assembly. This resulted in a total of eight sets of paired 1‐m^2^ quadrats per experimental plot—four sets close to the core and four sets far from the core. Within each 1‐m^2^ quadrat, we visually estimated plant cover (from 0.1% to 100%) of all species rooted within the quadrat. From these data we were able to quantify the richness and community composition of the entire seeded community, as well as focus on the subset of plant species that moved from the core.

### Statistical analysis

All statistical analyses were run in R version 4.0.3 (R Core Team, 2020). Data and code to recreate all analyses and figures are available in Zenodo (LLSullivan, 2022).

#### Target plant movement

To determine whether herbivores affected the distance the target species spread into the low‐diversity matrix, we used a generalized linear model with a gamma distribution because our data were not normally distributed owing to a heavy right skew. The response variable was the distance of each plant from the core, and the main effects were species and treatment (herbivores or no herbivores present), because no interaction was found (*p* = 0.21). We assessed the significance of the main effects using Wald chi‐square distribution and Type III sums of squares with the Anova() function in the car package (Fox & Weisberg, 2018). We also explored the magnitude of the effect of herbivory on our target species and found some species were more influenced by herbivory than others (Appendix S1: Figure S2).

#### Community composition

We focused on the 16 species that were planted in the core and moved into the matrix, hereafter called “moved” species, because these are the species that show evidence of assembling into the local communities from the regional species pool. We analyzed how distance from the core (near vs. far), herbivores, and competition (low vs. high) affected alpha diversity (richness) and community composition. For alpha diversity, we examined both the moved species and the species seeded in the matrix but excluded nonseeded species, which made up a small proportion of species and were uniformly distributed. Here we used a linear mixed‐effects model with alpha diversity of the moved species, all planted species as the response variable, and the additive effect of herbivores, dispersal distance, and competition status as our predictor variables (no significant interactions were found: *p* = 0.96), with transect nested within plot as a random intercept. We assessed the significance of these main effects using a Wald chi‐square distribution with Type III sums of squares. Then, to explore how our treatments altered the community composition of the moved species, we used a multivariate analysis with the vegan package (Oksanen et al., 2015). We explored both presence‐/absence‐based and cover‐based composition metrics, but both metrics provided qualitatively similar results, so we report only the presence‐/absence‐based metrics here. We transformed our data to reflect the presence or absence of each species within each quadrat and then created a distance matrix using Jaccard dissimilarity to examine the community of our moved species. We ran permutational multivariate analysis of variance (PERMANOVA), and modeled how the additive effects of our predictor variables (competition, dispersal, and herbivory) influenced the dissimilarity matrix for each plot, with the random effect of plot accounted for with strata, and ran our analysis for 999 permutations. We visualized our results using nonmetric multidimensional scaling with *k* = 3 dimensions (produced <10% stress) to ordinate the plant communities.

#### Species trade‐offs

Finally, to explore potential trade‐offs in ability to establish relative to our three process axes, we calculated a ratio of occurrence between quadrats paired by each process, then visualized these ratios in a three‐dimensional (3D) figure. To explore how herbivory influenced the establishment of the moved species, we created an occurrence ratio by dividing the number of herbivore quadrats in which a moved species was present by the number of herbivore‐excluded quadrats in which a species was present. A herbivore occurrence ratio >1 demonstrated that herbivores promoted the establishment of that species, whereas a ratio <1 indicates that herbivores suppressed establishment. A ratio of 1 indicates that the species is equally likely to establish in quadrats with or without herbivores present. For the competition occurrence ratio, we divided the number of high‐competition (uncovered) quadrats in which a species was found by the number of low‐competition (covered) quadrats in which it was found; values >1 indicate a positive establishment response to competition. Finally, the dispersal occurrence ratio examined the ratio of species occurrence in far quadrats divided by that in near quadrats; a value >1 indicates more frequent occurrence in far quadrats. For all occurrence ratios, if a species did not occur in any quadrat within a particular treatment (e.g., a given species was never found in a far quadrat), we replaced the 0 within the value 0.01, which is less than the probability that a species occurred in 1 of the 64 total quadrats, in order to obtain a number for plotting. We additionally created a correlation matrix of occurrence data (Appendix S1: Figure S5).

## RESULTS

### Target plant movement

**FIGURE 2:**
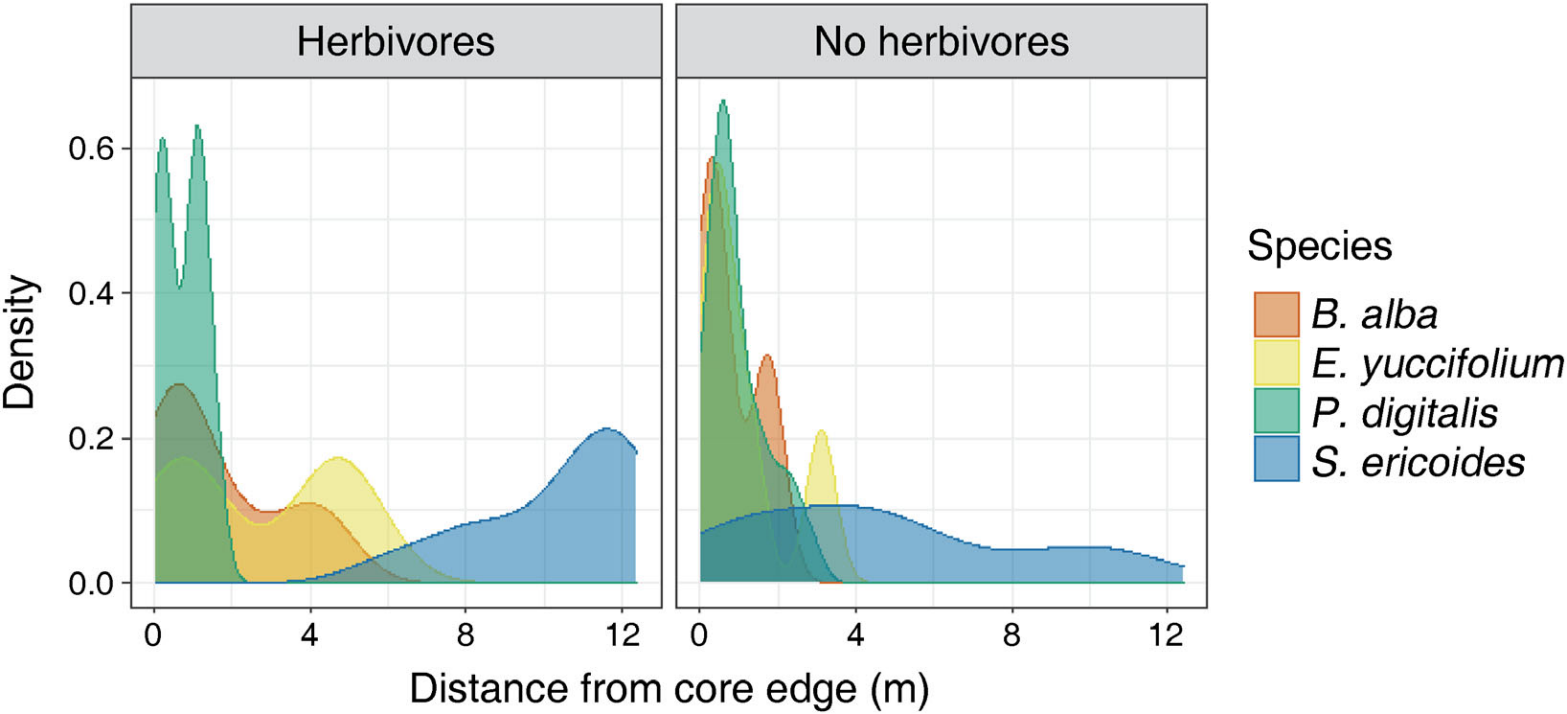
Movement of *Baptisia alba*, *Eryngium yuccifolium*, *Penstemon digitalis*, and *Symphyotrichum ericoides* in the presence and relative absence of herbivores.

Our four target plant species differed in how far they spread from the central core (χ^2^_df=3_ = 113.31; *p* < 0.0001), and this distance increased (across all four species) with herbivore presence (χ^2^_df=1_ = 7.20; *p* = 0.007; Figure 2; Appendix S1: Figure S3). In general, in the presence of herbivores, more individuals tended to move farther, with individuals in the farthest 75%–99% of the distribution moving 8.1–12.3 m with herbivores versus 2.4–10.7 m without herbivores (Appendix S1: Figure S3). Despite no significant interaction between species and herbivores (*p* = 0.21), there were subtle species‐specific responses. *S. ericoides*, which produces tufted achenes, moved farther than the other species, and this pattern showed a strong increase under herbivory. *B. alba* and *E. yuccifolium* showed similar increases in movement with herbivore presence, but at shorter distance scales, whereas *P. digitalis* was the only species that moved less when herbivores were present (Figure 2; Appendix S1: Table S1).

### Community composition

**FIGURE 3:**
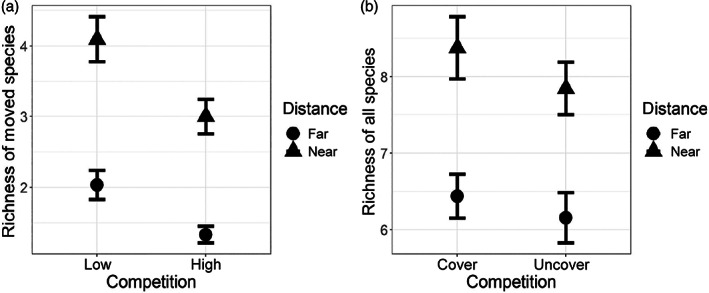
Effects of competition (covered represents low competition) and dispersal distance on alpha diversity (richness) of species in each quadrat. (a) Species that moved into matrix from core. (b) All species, including seeded and moved species.

Herbivores did not significantly affect the alpha diversity (richness) of the 16 moved species (χ^2^_df=1_ = 1.97; *p* = 0.16); however, the distance from the core (χ^2^_df=1_ = 137.11; *p* < 0.0001) and competition (e.g., high vs. low competition) (χ^2^_df=1_ = 32.83; *p* < 0.0001) significantly increased the quadrat richness of these moved species. Quadrats closer to the core had higher alpha diversity than those far from the core, and quadrats that were initially covered (low competition) had higher alpha diversity of moved species than those that were seeded with the initial restoration (Figure 3). When we examined the alpha diversity of all planted species, we found similar results. Only distance from the core had a significant effect on the species richness, with closer quadrats having higher alpha diversity than farther quadrats (χ^2^_df=1_ = 37.95; *p* < 0.0001). When considering the community of the moved species, distance from core (*F*_df=1_ = 2.18; *p* = 0.001), herbivory (*F*_df=1_ = 1.98; *p* = 0.001), and competition (*F*_df=1_ = 0.80; *p* = 0.002) all influenced community composition (using Jaccard dissimilarity) (Appendix S1: Figure S4).

### Species trade‐offs

**FIGURE 4:**
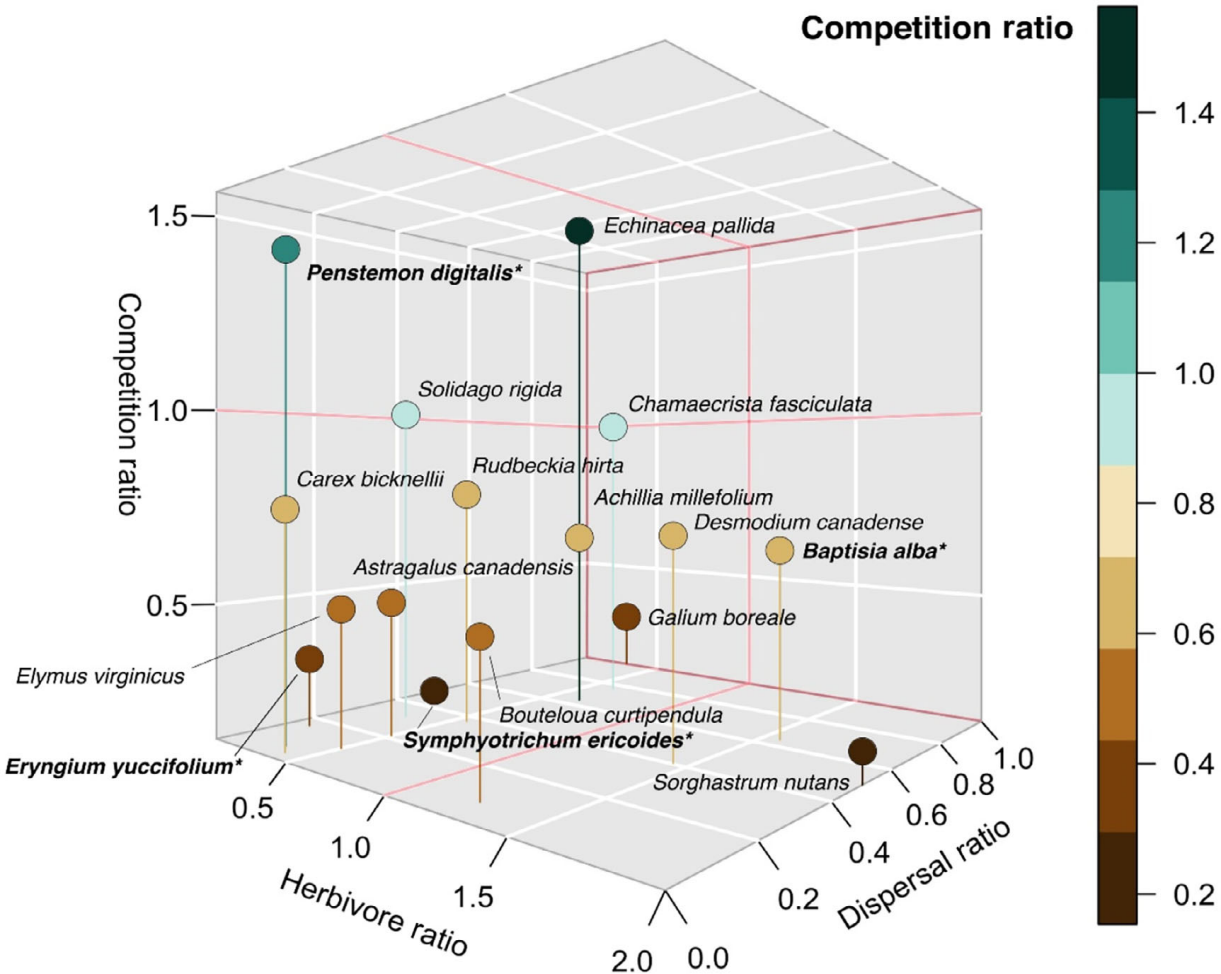
Individual species vary in their interactions with dispersal distance, competition, and herbivory. Bolded points with an asterisk indicate our four target species. For each axis, we calculated the ratio of the number of quadrats in which a species was found to calculate their likelihood of being affected by a process. Species with a herbivore ratio >1 suggests herbivores promote establishment. Species with a competition ratio >1 suggests competitors promote establishment (e.g., competition is less of a barrier to establishment). There are no species with a dispersal ratio >1, indicating that species are more likely to establish in near quadrats than far quadrats, but the ratio still indicates the degree to which they disperse longer distances (e.g., *Gallium boreale* is in nearly as many far quadrats as near quadrats, but *Eryngium yuccifolium* is much more likely to be in near quadrats). A ratio of 1 means the species was equally likely to establish in either of the treatment quadrats for that axis.

When visualizing the moved species in a 3D trade‐off space, each species is represented by a point indicating varying susceptibility to competition, herbivory, and dispersal (Figure 4). There is modest evidence for a trade‐off between herbivory resistance and competitive ability (*r* ^2^ = −0.15, Appendix S1: Figure S5) but a modest positive correlation between herbivory resistance and their dispersal ability (*r* ^2^ = 0.19; Appendix S1: Figure S5).

## DISCUSSION

Our work demonstrates the influence of dispersal on community assembly, both in isolation and in combination with competition and herbivory. Dispersal distance, competition, and herbivory all influenced the number and identity of species that joined local communities from the regional species pool, but when and where those processes were acting depended on the metrics examined and the scale of observation. By tracking the spread of species away from where they were initially seeded, we found inter‐ and intraspecific differences in effective dispersal patterns and that herbivores generally increased the distance at which individuals established and the overall abundance of individuals (Figure 2; Appendix S1: Figure S2, Table S1). These results indicate that variation in dispersal and the presence of herbivores affect community assembly processes (Neubert & Caswell, 2000). When we examined the alpha diversity of moved species in quadrats that varied in distance from the source population, we found that dispersal and competition mattered—quadrats closer to the source had higher species richness than those farther away, and more core species established in the low‐competition quadrats than the high‐competition quadrats, suggesting potential priority effects caused by the seeded plant community (Figure 3). Herbivores, on the other hand, did not significantly affect species richness (Figure 3). When we examined the composition of the community of moved species, we found that dispersal, competition, and herbivory had additive effects, with no significant interactions between them (Appendix S1: Figure S4). While there is inter‐ and intraspecific variation in dispersal, competitive ability, and tolerance of herbivory, most plant species visually demonstrated trade‐offs (Figure 4), leading to different communities in different spatial and biotic contexts.

By examining the spatial patterns of movement relative to the source population in the core, we demonstrated that species varied in effective dispersal and that herbivores tended to increase the distance at which plants established (Figure 2). Traditionally, our understanding of community assembly often ignores or simplifies species differences in dispersal (HilleRisLambers et al., 2012). However, our results showed that there was interspecific variation in establishment (Figure 2), likely owing to differences in dispersal strategy. *S. ericoides* and *E. yuccifolium* both contain structures that facilitate wind dispersal, consistent with their moving farther, whereas *B. alba* and *P. digitalis* demonstrate gravity or explosive dispersal, limiting movement distance. In addition to interspecific differences, we also saw intraspecific differences that depended on herbivore presence. Specifically, a greater proportion of *S. ericoides*, *E. yuccifolium*, and *B. alba* individuals were found farther from the core when herbivores were present, whereas *P. digitalis* individuals were found closer to the source population in the presence of herbivores, although these species differences were not significant. These results mirror previous studies that showed that herbivores could both decrease (Sullivan et al., 2016) and increase (Vellend et al., 2003) dispersal distance in population‐based studies. The mechanism for the interaction between herbivory and dispersal could either be direct (e.g., herbivores move seeds or otherwise prevent establishment [through herbivory or trampling]) or indirect (e.g., herbivores affect physical structure of the environment to increase or decrease dispersal or reduce the abundance and chance of rare, longer‐distance dispersal events). Future studies examining seed movement directly would be desirable to disentangle these possibilities and in different settings. Our results demonstrate the importance of monitoring the spread of species to reveal both inter‐ and intraspecific differences in effective dispersal, since scaling up to examine community‐level patterns on a quadrat scale can miss rare but important patterns in movement. We suggest that mean dispersal distance modeling should include a measure of variance caused by intraspecific variation between species and contexts (e.g., hierarchical Bayes models) because plant movement is species‐ and context‐dependent (Sperry et al., 2019).

Dispersal, competition, and herbivory collectively affect which species will join existing local communities (Appendix S1: Figure S4). In our study, dispersal limitation and herbivory had the strongest effects on local community composition, followed by competition. The number of species that spread from the core declined significantly with distance, with higher species richness in quadrats near the source population, as might be expected. However, although herbivores affected community composition, they did not significantly affect the alpha diversity within our sampling quadrats, which suggests turnover in the plant species present between herbivore and no‐herbivore plots. On the other hand, competition, which had the weakest effect on community composition in our experiment, had a strong effect on species richness, with more species establishing in the areas with reduced competition. It appears that existing species in quadrats with high competition prevented the establishment of newly arriving species (Dickson et al., 2012). Priority effects may have caused these establishment differences because the uncovered quadrats (high competition) were dominated by species from the initial low‐diversity seed addition, whereas the covered quadrats (low competition) had far fewer species that were seeded into the matrix and greater establishment by species dispersing from the core. Thus, open space, free from priority effects by dominant species, appears to be important for rare establishment events to occur.

Species trade‐offs are also likely to influence community assembly processes since few species thrive in all biotic and abiotic environmental contexts (Figure 4). For example, a species that is a strong competitor is less likely to also be a good disperser and tolerant of herbivory (Tilman, 1990). We found evidence for trade‐offs in how species respond to dispersal, competition, and herbivory assembly processes in our study. There was a modest negative correlation between species that were able to establish in plots with high herbivory and competition (*r* ^2^ = −0.15) (Appendix S1: Figure S5). For example, *P. digitalis* was a strong competitor but was more susceptible to herbivory, which is consistent with the competition–defense trade‐off. This trade‐off should, in theory, reduce the ability of *P. digitalis* to exclude other species in the presence of herbivory (Viola et al., 2010). Interestingly, we also found a modest positive correlation between species that could disperse farther and could establish in the presence of herbivores (*r* ^2^ = 0.19) (Appendix S1: Figure S5). This result from our 1‐m^2^ quadrats matched our target species results, where species’ dispersal ability increased in the presence of herbivores (Figure 2). For example, *Physostegia virginiana* and *Achillea millefolium* were both able to disperse relatively more frequently to far plots and were more resistant to herbivory. Ultimately, no species was able to disperse far, compete strongly, and tolerate herbivory at the same time, although a few species (e.g., *E. yuccifolium*) seemed to perform poorly along all three axes (Figure 4). These results are unsurprising and provide evidence that species have evolved different life history strategies and are constrained in their abilities to respond optimally to all assembly processes (Stearns, 1989). These relationships help understand the mechanisms that can lead to overall community composition and structure. Explicitly including trade‐offs should improve models' ability to predict the composition of communities (Tilman, 1990).

Accurately predicting community assembly is central to understanding how communities may behave in future scenarios under global change and as ecosystem restoration becomes more common to repair highly degraded systems. Here we demonstrate the value of focusing on variation between individual species and understanding how species fit together into communities. Also, explicitly incorporating dispersal into community assembly and recognizing the interactions between dispersal and establishment and other processes such as competition and herbivory are likely to improve our understanding of why communities differ locally. Prairie (and other) restorations often overcome initial dispersal limitations through seeding, but this strategy may not optimize community outcomes owing to trade‐offs between dispersal and other processes. Given that we found alpha diversity was greater in quadrats that were not seeded, including “safe” sites that allow natural dispersal with proximity to an adequate source population may provide better restoration outcomes, as opposed to relying solely on seed additions (Grygiel et al., 2014). Incorporating both high‐diversity seed additions and “safe” sites for natural dispersal to occur in restorations may be the next step to combat diversity loss in restorations. Although we focused on a few key interactions between dispersal, competition, and herbivory, additional research on the unexplored interactions among these and other processes is needed to understand how and when interspecific variation in trade‐offs between dispersal and other biotic or abiotic processes affects community assembly.

## CONFLICT OF INTEREST

The authors declare no conflict of interest.

## Supporting information


Appendix S1

## Data Availability

Data and code (LLSullivan, 2022) are available in Zenodo at https://doi.org/10.5281/zenodo.6799600.
