# Supplementary material for: The effects of dispersal, herbivory, and competition on plant community assembly
Source: Ecology. 2022 Nov 10;104(1):e3859. doi: 10.1002/ecy.3859 (PMC10078099; doi:10.1002/ecy.3859)
Supplement: Supplementary file 1 — Appendix S1 [file ECY-104-0-s001.pdf]

Samantha A. Allbee, Haldre S. Rogers, Lauren L. Sullivan. The effects of dispersal, herbivory, and competition on plant community assembly. Ecology.

Appendix S1

Restoration details, experimental design, target species descriptions, and species list:

Oakridge Research and Education Prairie (Oakridge) is located in the center of town in Ames, IA. This site was rotating corn and soy for over 100 years before we restored the prairie in 2012. The year before seeding, the site was in corn. We tilled the ground once in the previous fall (2011) and once before site construction in February 2012. Before seeding, we constructed all plot infrastructure (See main text Fig 1) including: designating core and matrix areas, building herbivore exclusion fences, and placing cover strips on the ground. Additionally, the prairie is bordered by an agricultural field with rotating corn/soy crops, a forested area, and neighborhoods on two sides; the nearest prairie (reconstructed or remnant) is more than 1 mile away.

Design of herbivore exclusion fences is detailed in Mortensen et al. (2013). The four herbivore exclusion fences excluded white-tailed deer (*Odocoileus virginianus*), and prairie voles (*Microtus ochrogaster*). Deer were excluded with a three-strand electric fence, with two strands running approximately 38.1 and 101.6 cm above ground level that are both set approximately 1m outside a third strand running 76.2 cm above ground level. This design discourages entry by deer as they cannot jump the horizontal distance of the two fences. The inner fence also consists of 60.96 cm tall hardware cloth at the base that extends 30.48 cm belowground to exclude voles. Around this hardware cloth, we mowed a 1m strip around the cloth to create a "fear strip" that discouraged voles from crossing into the plots. In the summer of 2012 and 2013 we trapped small mammals from inside the fences and removed the voles when they were found inside in order to keep vole population densities low. We did not remove mice, as they had no problem climbing the fence or crossing the fear strips, and thus we could not exclude them with this experimental design. Fencing reduced the population size of voles (Mortensen et al. 2013), and the number of voles removed from fenced areas can be found in the supplement of Mortensen et al. (2018).

In March 2012, we seeded Oakridge at a density of ~4.48 seeds/m^2 (~65% forbs and ~35% grass by weight). Core areas (circular areas with a 19.2 m diameter, 0.28 ha) received 51 species and were nested inside each of eight 32x32m, 0.10 ha blocks. Within each block, outside of the high diversity core area we planted 14 species in this "low diversity matrix", and the 14 matrix species were a subset of the 51 from the high diversity core. The number of species used in the low diversity matrix approximates diversities in most prairie restorations, while the high diversity core approximates diversities in most prairie remnants (e.g Martin, Moloney, & Wisley, 2005). In addition, species in the low-diversity matrix were selected to represent all typical functional groups (C3/C4 grass, forb, legume). See species list on the next page. Seeds for the prairie were purchased from Allendan Seed Company (https://www.allendanseed.com) and Prairie Moon Nursery (https://www.prairiemoon.com). All areas were seeded by broadcast seeding bags of seed mixed with sand to help keep seeds suspended equally and stomped down manually to encourage good seed-soil contact. All seeders wore removable plastic booties when stomping, and removed them between areas (e.g., changed between blocks and between core and matrix areas) to avoid seed contamination.

It was unfortunately too expensive to have a separate battery for each fence and have the fenced and unfenced plots interspersed. Thus, we needed to keep two fenced plots adjacent for feasibility of the experiment. In addition, two plots were not contiguous with the other six, because we needed to avoid a depression in the field to keep the abiotic environment consistent

and could not fit all eight plots in the same area. We do not believe the location of the fenced plots influenced community assembly. To test this assumption, we examined the composition of plant cover in all plots in the first year after seeding (as everything was establishing), and between fenced plots in the year we conducted this experiment (2019). We found no differences between species composition across both fenced and unfenced plots in the first year of seeding (PERMANOVA of matrix [$p = 0.458$] and core [$p = 0.235$] composition differences among plots). This provides the strongest evidence that the no-herbivore and herbivore plots are experiencing similar conditions. In addition, we found no differences between the four no-herbivore plots in 2019 (PERMANOVA with just fenced plots found no effect of plot [$p = 0.187$]). This convinced us that community composition was similar in the four fenced plots the year we conducted the experiment, so the two non-contiguous plots are performing similarly to the two contiguous plots. Thus, that gives us some reassurance that the non-interspersed nature of the plots was not having a strong effect on the outcome of our experiment.

Target species descriptions

Penstemon digitalis is a clump forming perennial that produces dark brown and oval seed capsules that contain a large number of seeds. The inflorescence eventually falls over which helps distribute the seeds and can be carried by wind for short distances (Gleason and Cronquist 1963). *Baptisia alba* produces seed pods that burst open and disperse explosively (Gleason and Cronquist 1963). *Symphyotrichum ericoides* is a perennial forb that produces achenes which have tufts to help them blow away in the wind (Gleason and Cronquist 1963). *Eryngium yuccifolium* is a perennial forb with flowerheads that are subtended by pointed bracts. Each produces two fruits (schizocarps) which have serrate wings for dispersal in the wind (Gleason and Cronquist 1963).

References

Gleason, H. A., and A. Cronquist. 1963. Manual of vascular plants of northeastern United States and adjacent Canada. van Nostrand Princeton, NJ.

Martin, L.M., Moloney, K.A., and B.J. Wisley. 2005. An assessment of grassland restoration success using species diversity components. Journal of Applied Ecology 42: 3

Mortensen, B., Danielson, B., Harpole, W.S., Alberti, J., Arnillas, C.A., Biederman, L., Borer, E.T., Cadotte, M.W., Dwyer, J.M., Hagenah, N. and Y. Hautier. 2018. Herbivores safeguard plant diversity by reducing variability in dominance. Journal of Ecology, 106:1

Mortensen, B. D., L. L. Sullivan, and W. S. Harpole. 2013. Oakridge Research and Education Prairie. Iowa State University Research and Demonstration Farms Progress Reports 2012(1). https://www.iastatedigitalpress.com/farmreports/article/id/5383/

Oakridge Species List

Remember: The high diversity cores have all of the species and are in the circles in the center of each block. The low diversity matrix is a subset of the core species and are seeded all over the rest of the field.

**High Diversity Core**			**Low Diversity Matrix**	
**Species**	**Common Name**		**Species**	**Common Name**
**FORBS**			**FORBS**	
Achillea millefolium	Yarrow		*Anemone cylindrica*	Candle Anemone
Anemone cylindrica	Candle Anemone		*Asclepias incarnata*	Swamp Milkweed
Anemone virginiana	Tall Thimble Weed		*Aster laevis*	Smooth Blue Aster
Asclepias incarnata	Swamp Milkweed		*Brickellia eupatorioides*	False Boneset
Asclepias sullivantii	Sullivan's Milkweed		*Lespedeza capitata*	Roundheaded Bushclover
Asclepias tuberosa	Butterfly Milkweed		*Liatris pychnostachya*	Prairie Blazing Star
Asclepias verticillata	whorled milkweed		*Dalea purpurea*	Purple Prairie Clover
Aster azureus	Sky Blue Aster		*Ratibida pinnata*	Gray-headed Coneflower
Aster laevis	Smooth Blue Aster		*Zizia aurea*	Golden Alexander
Astragalus canadensis	Canada Milk Vetch			
Baptisia alba	Wild White Indigo		**GRASSES**	
Cassia fasciculata	Partridge Pea		*Andropogon gerardii*	Big Bluestem
Desmodium canadense	Showy Tick Trefoil		*Carex vulpinoidea*	Fox Sedge
Echinacea pallida	Purple Coneflower		*Elymus canadensis*	Canada Wild Rye
Eryngium yuccifolium	Rattlesnake Master		*Schizachyrium scoparium*	Little Bluestem
Euphorbia corollata	Flowering Spurge		*Sporobolus clandestinus*	Rough Dropseed
Galium boreale	Northern Bedstraw			
Heuchera richardsonii	Prairie Alumroot			
Brickellia eupatorioides	False Boneset			
Lespedeza capitata	Roundheaded Bushclover			
Liatris aspera	Rough Blazing Star			
Liatris pycnostachya	Prairie Blazing Star			
Lobelia cardinalis	Cardinal Flower			
Lobelia siphilitica	Great Blue Lobelia			
Penstemon digitalis	Foxglove Penstemon			
Dalea candida	White Prairie Clover			
Dalea purpurea	Purple Prairie Clover			
Physostegia virginiana	Obedient Plant			
Potentilla arguta	Tall/Prairie Cinquefoil			
Ratibida pinnata	Gray-headed Coneflower			
Rudbeckia hirta	Blackeyed Susan			
Solidago rigida	Rough Goldenrod			
Solidago speciosa	Showy Goldenrod			
Symphyotrichum ericoides	Heath Aster			
Verbena stricta	Hoary Vervain			
Vernonia fasciculata	Ironweed			
Veronicastrum virginicum	Culver's Root			
Zizia aurea	Golden Alexander			

**GRASSES**				
Andropogon gerardii	Big Bluestem			
Schizachyrium scoparium	Little Bluestem			
Bouteloua curtipendula	Sideoats Grama			
Carex bicknellii	Bicknell's Sedge			
Carex brevior	Shortbeak Sedge			
Carex vulpinoidea	Fox Sedge			
Elymus canadensis	Canada Wild Rye			
Elymus virginicus	Virginia Wild Rye			
Sorghastrum nutans	Indian Grass			
Sporobolus clandestinus	Rough Dropseed			
Sporobolus heterolepis	Prairie Dropseed			
Stipa (Heterostipa) spartea	Porcupine Grass			
**UNIQUE ASTERACEAE (e.g., seeded only in plot in parentheses")**				
Silphium laciniatum	Compass Plant (1)			
Helianthus maximiliana	Maximillian Sunflower (2)			
Helianthus pauciflorus	Rigid Sunflower (3)			
Heliopsis helianthoides	Oxeye/False Sunflower (4)			
Helianthus grosseserratus	Sawtooth Sunflower (5)			
Symphyotrichum novae-angliae	New England Aster (6)			
Eupatorium perfoliatum	Common Boneset (7)			
Rudbeckia subtomentosa	Sweet Black-eyed Susan (8)			

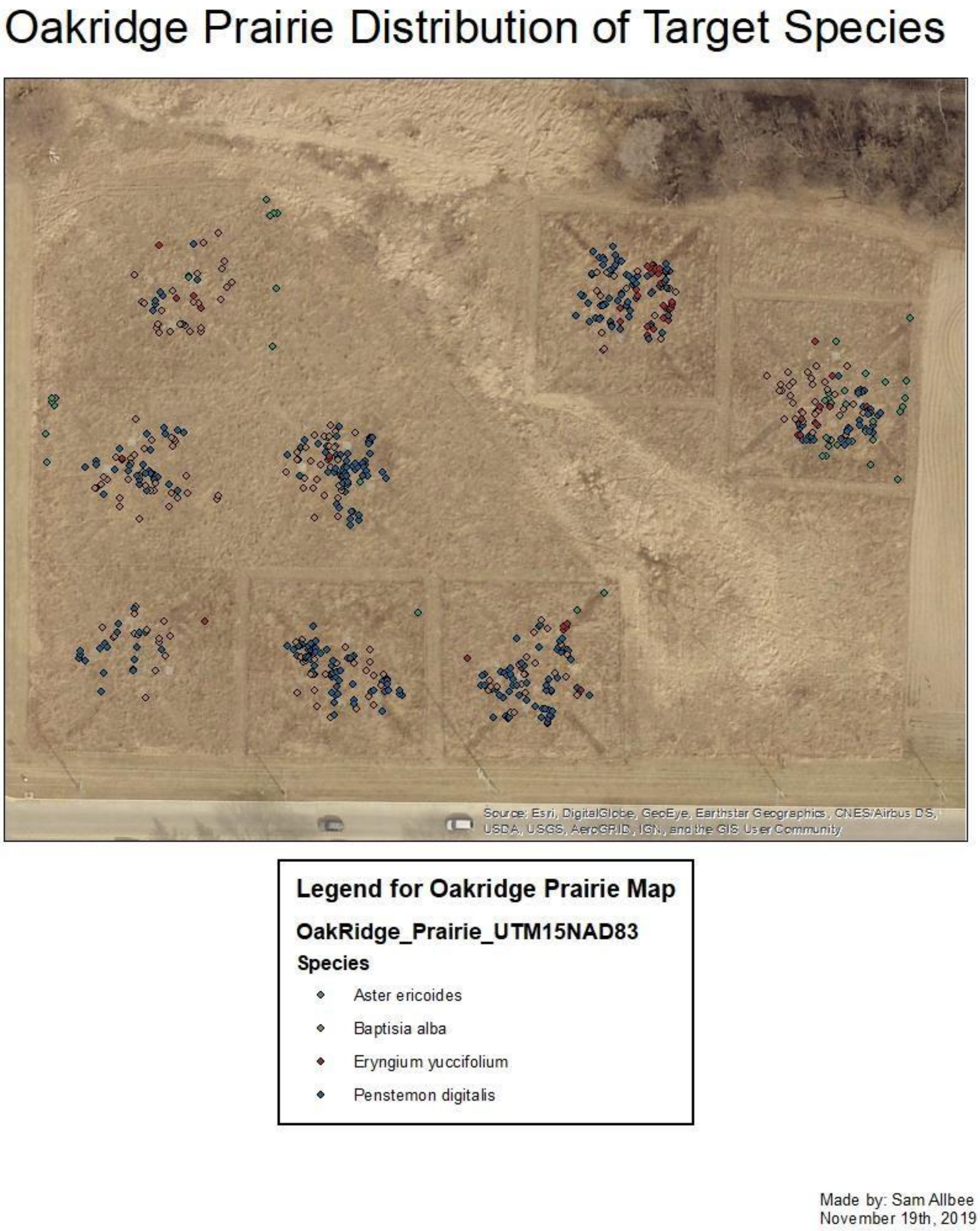

Figure S1: GPS locations of all individuals found of target species across the eight experimental plots.

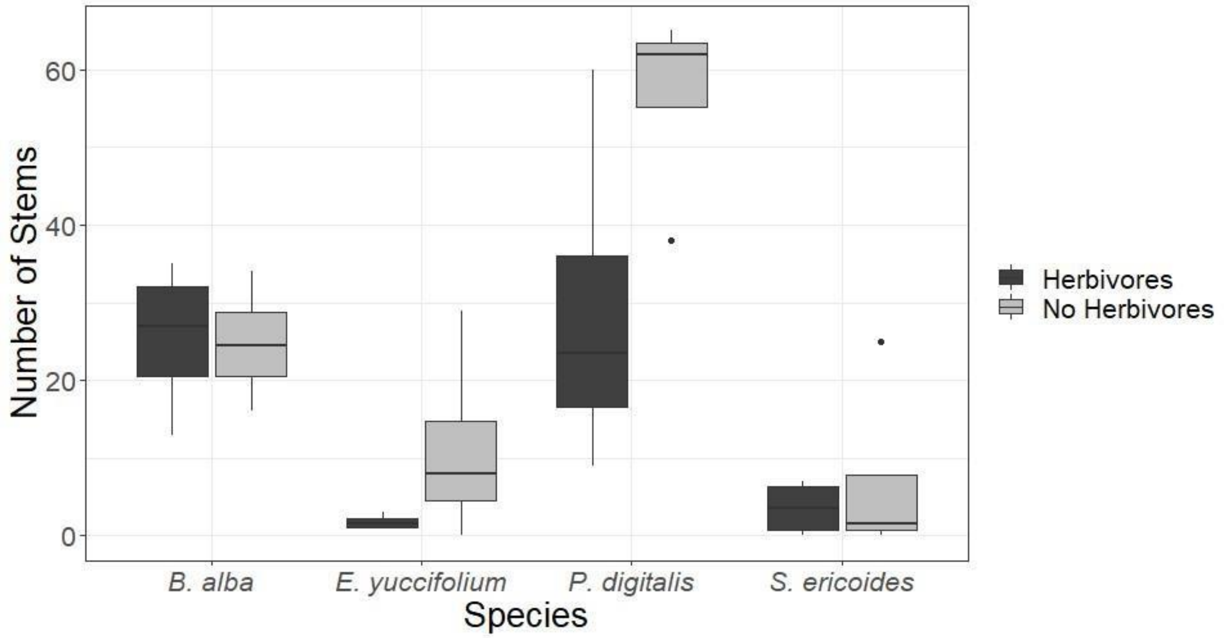

Figure S2: Herbivore effects on plant abundance of *B. alba*, *E. yuccifolium*, *P. digitalis*, and *S. ericoides.* To assess whether herbivores affect the abundance of stems of our target species within each plot, we used a generalized linear mixed effects model with a negative binomial distribution to accurately convey the overdispersion in stem count data with the glmer.nb() function in the lme4 package. Our response variable was total number of stems, and our predictor variables were the interaction between species identity and herbivore treatment. We included plot as a random intercept. We again assessed the significance of main and interactive effects using the Wald Chisquare distribution and Type III sums of squares. Both herbivore presence ($x^2_{df=1}$=5.53; $p = 0.019$) and species ($x^2_{df=3}$=38.93; $p < 0.0001$) influenced the stem abundance of our four target species. Overall species differed from each other in the number of stems found in the matrix (*P. digitalis* had the most stems, then *B. alba*, then *E. yuccifolium* and *S. ericoides*), and herbivores decreased the number of stems.

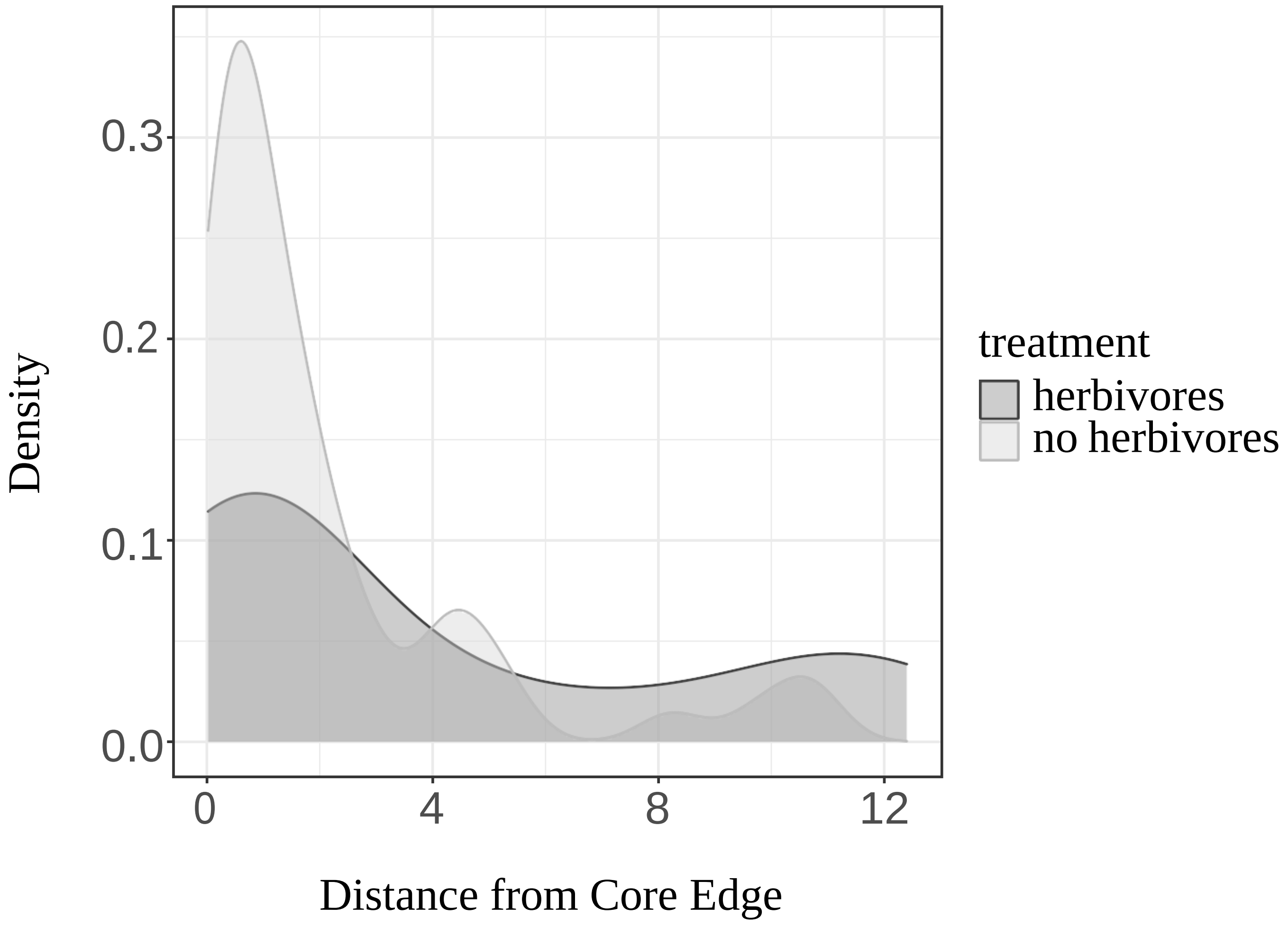

Figure S3: The distance four focal plant species spread from the central core by herbivore presence. In the presence of herbivores more individuals tended to move further, with individuals in the farthest 75-99% of the distribution moving 8.1 - 12.3m with herbivores versus 2.4 - 10.7m without herbivores.

Table S1: The distances (m) each species moved at 1%, 25%, 50%, 75% and 99% of their moved distribution with and without herbivores. Species-specific responses to the treatments were often subtle. *S. ericoides*, which produces tufted achenes, moved farther than the other species, and this pattern showed a strong herbivore effect. In the presence of herbivores, *S. ericoides* established at farther distances across its entire movement distribution (6-12 m) than when herbivores were absent (1-10m). *B. alba* and *E. yuccifolium* showed similar increases in movement with herbivore presence, but at shorter distance scales. *B. alba* individuals in the farthest 25-99% of the distribution moved 0.5-4.3m when herbivores were present versus 0.3-1.7m when herbivores were absent. *E. yuccifolium* individuals in the farthest 25-99% of the distribution moved 1.7-4.7m when herbivores were present, versus 0.5-3m when herbivores were absent. *P. digitalis* was the only species that moved less when herbivores were present, with individuals in the farthest 25-99% of the distribution moving 0.2-1.6m when herbivores were present, versus 0.5-2.7m when herbivores were absent.

**Species**	**Treatment**	**1%**	**25%**	**50%**	**75%**	**99%**
B. alba	herbivores	0.085	0.509	0.790	2.630	4.275
	no herbivores	0.163	0.305	0.454	1.096	1.713
E. yuccifolium	herbivores	0.783	1.736	2.729	3.722	4.676
	no herbivores	0.159	0.496	0.579	1.275	3.039
P. digitalis	herbivores	0.047	0.223	0.973	1.107	1.558
	no herbivores	0.029	0.526	0.687	1.390	2.747
S. ericoides	herbivores	6.316	9.064	11.179	11.972	12.363
	no herbivores	0.110	1.557	4.290	6.057	10.741

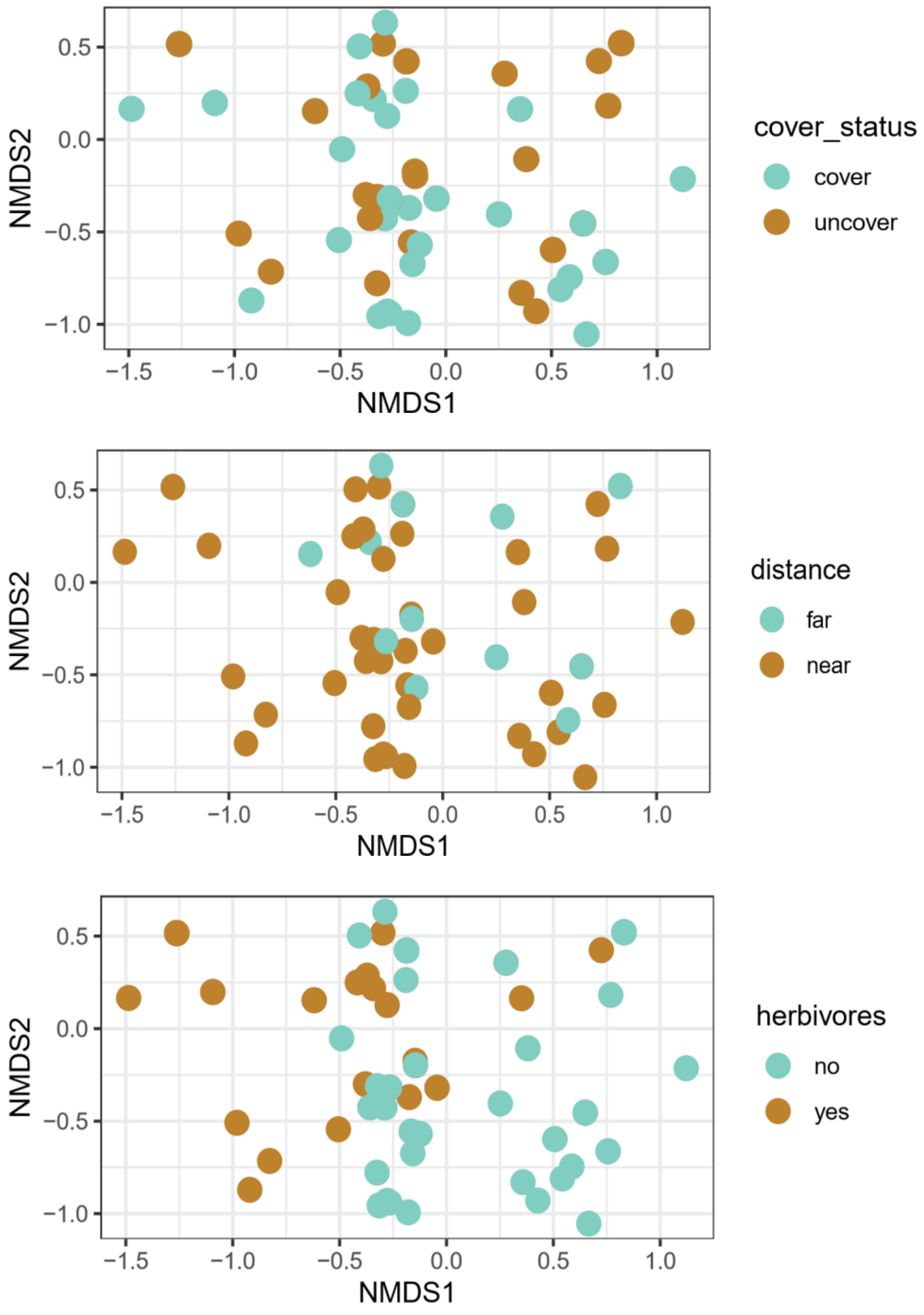

Figure S4: Competition (cover – low, uncover - high), dispersal distance from core (near and far quadrats), and herbivory (yes and no) all influenced community composition of the moved species.

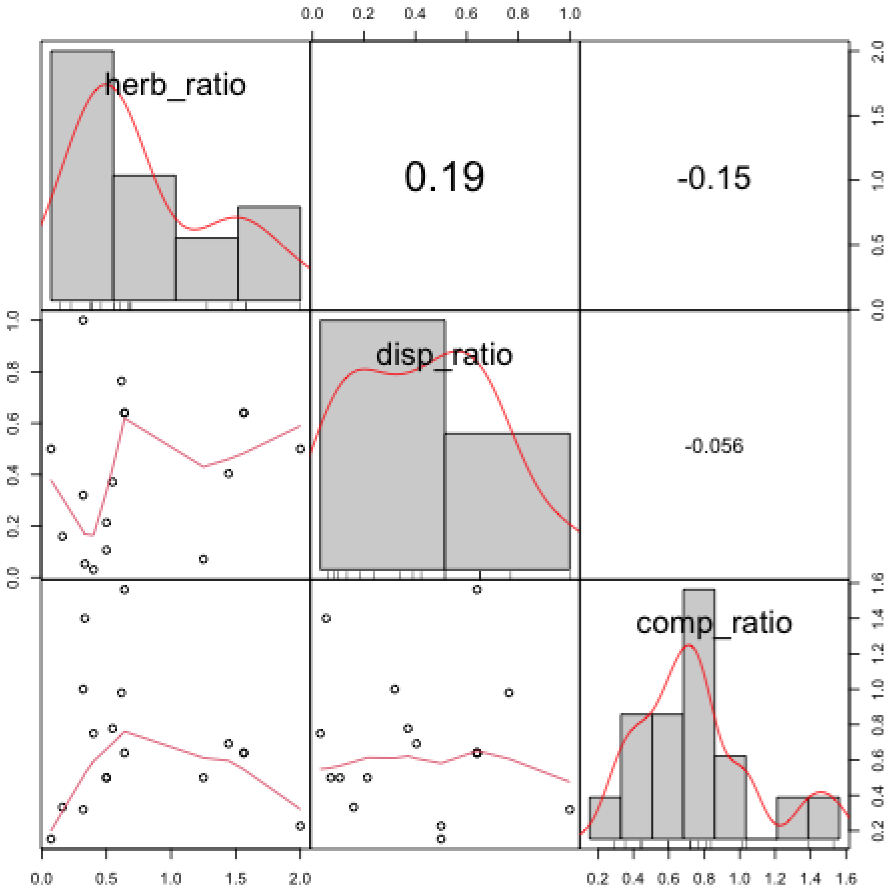

Figure S5: Correlation matrix of occurrence data to determine how species trade-off between competition, herbivory, and dispersal. We found a modest positive correlation (r2 = 0.19) between species that were able to establish in herbivore plots and dispersal plots. This indicates that species that are establishing in farther plots are also establishing in plots with higher herbivore pressure. This backs up the findings that herbivores increase dispersal ability (Fig. 2). We also found a modest negative correlation (-0.15) between species that were able to establish in herbivore plots vs high competition plots, indicating a trade-off. Species that were able to establish under stronger competition were not able to establish under higher herbivore pressure. While these correlations were small, we believe the indicate the potential for these species to trade-offs between these different assembly drivers.
